# National Analysis of Emergent Reoperations After Metabolic Bariatric Surgery Over 6 Years

**DOI:** 10.1097/AS9.0000000000000611

**Published:** 2025-09-23

**Authors:** Tina Bharani, Divyansh Agarwal, Alec Beekley, Malcolm Robinson

**Affiliations:** From the * Department of Surgery, Thomas Jefferson University Hospital, Philadelphia, PA; † Department of Surgery, Massachusetts General Hospital, Boston, MA; ‡ Department of GI Surgery, Brigham and Women’s Hospital, Boston, MA.

## Abstract

**Objective:**

Discuss rates of emergency reoperations within 30 days after metabolic bariatric surgery (MBS) from 2017 to 2022 in Metabolic and Bariatric Surgery Quality Improvement Program (MBSAQIP) Centers of Excellence. Also, identify the preoperative factors contributing to increased risk of emergency reoperation after MBS and evaluate the impact of emergent reoperations on postoperative outcomes within 30 days.

**Background:**

While prior studies have evaluated the outcomes associated with revisional surgeries many years after index MBS and reoperations within 30 days, little is known about the trends in emergent reoperations after MBS.

**Methods:**

MBSAQIP database from 2017 to 2022 was utilized for evaluating the trends and outcomes related to emergent reoperations after MBS.

**Results:**

Overall, the rate of emergency reoperations has declined significantly (from 0.84% to 0.60%). Up to half the emergency reoperations occurred within postoperative day 0–3 and two-thirds occurred within 7 days from surgery. Some of the preoperative comorbidities and demographics of patients requiring emergency reoperations were older age, males, history of deep venous thrombosis, history of myocardial ischemia, gastroesophageal reflux disease, therapeutic anticoagulation, and chronic obstructive pulmonary disease. Analysis of postoperative outcomes after matching patients who underwent emergency reoperation with those who did not undergo reoperation showed higher rates of death (3.48% vs 0.19%, *P* < 0.001), acute renal failure (3.4% vs 0.1%, *P* < 0.001), unplanned ICU admissions (30.83% vs 0.83%, *P* < 0.001) and organ space infections (21.32% vs 0.52%, *P* < 0.001).

**Conclusions:**

Emergent reoperations after MBS have significantly worse morbidity and mortality. Recognition of high-risk preoperative factors can help optimize the modifiable factors preoperatively and facilitate closer monitoring of vulnerable patients.

## INTRODUCTION

Metabolic bariatric surgery (MBS) remains the most effective and durable treatment for obesity and its associated metabolic comorbidities.^1,2^ The number of MBS performed in the United States over the past 6 years has risen from 228,005 in 2017 to 279,967 in 2022, with a nadir in 2020 due to COVID pandemic.^3^ The perioperative safety of bariatric surgery also continues to improve over the years with mortality rates ranging from 0.03% to 0.2%, which is comparable to that of laparoscopic cholecystectomy.^4–6^ However, these surgeries are associated with 3% to 6% risk of major complications within 30 days, and 1% to 3% require reoperations.^6–10^

Some of the common reasons for reoperation identified in prior studies include hemorrhage, anastomotic or suture line leaks, intestinal obstructions, and adjacent organ injury.^11,12^ Reoperation after MBS has been associated with significant morbidity and increased risk of mortality within 30 days.^11^ While the short-term perioperative outcomes in patients requiring reoperation are worse, the long-term outcomes showed no difference in weight loss and remission of comorbidities.^8^ Postoperative complications within 30 days after MBS requiring emergent reoperation pose significant risk on patients with obesity given their comorbidities, higher American Society of Anesthesiologists (ASA) score, and atypical profile of symptoms (example absence of peritonitis) causing delays in management.^12^ Postoperative complications requiring emergent reoperations may possess even higher risk for morbidity and mortality given the compromised state of the patient and the urgency of the intervention. Thus, recognizing and optimizing factors contributing to increased risk of emergent reoperations is necessary to mitigate the rates of reoperation and improve the outcomes after MBS.

While prior studies have evaluated the outcomes associated with revisional surgeries many years after index MBS and reoperations within 30 days, little is known about the trends in emergent reoperations after MBS. In this study, we discuss the rates of emergency reoperation within 30 days after MBS from 2017 to 2022 in Metabolic and Bariatric Surgery Quality Improvement Program (MBSAQIP) Centers of Excellence. We also identify the preoperative factors contributing to increased risk of emergency reoperation after MBS and evaluate the impact of emergent reoperations on postoperative outcomes within 30 days.

## METHODS

### Study Design and Setting

The MBSAQIP database from 2017 to 2022 was utilized for evaluating the trends and outcomes related to emergent reoperations after MBS. All the patients who underwent primary MBS from 2017 to 2022, including sleeve gastrectomy (SG), Roux-en-Y gastric bypass (RYGB), biliopancreatic diversion (BPD), and gastric band, and required emergency reoperation within 30 days were included in the study. Participants who underwent endoscopic approach were excluded from the analysis.

### Variables

In the reoperation within 30 days subset of the MBSAQIP database from 2017 to 2022, those who underwent emergent reoperation were selected for analysis. The trend in the rates of emergency reoperations was analyzed over 6 years of study period. The trends in days passed from index operation to emergency reoperation was also analyzed. Preoperative demographics and comorbidities associated with higher risk of emergency reoperation were analyzed. Subanalysis of preoperative factors associated with high risk of emergency reoperations was also analyzed for SG and RYGB. This was followed by a comparative analysis of postoperative outcomes in patients who underwent emergency reoperations versus those who did not require emergency reoperations. The postoperative outcomes analyzed included mortality within 30 days, acute renal failure, cardiac arrest, myocardial infarction, cerebrovascular accident, pneumonia, pulmonary embolism (PE), unplanned ICU admission, wound disruption, sepsis, *Clostridium difficile* infection, urinary tract infection, and dehydration requiring treatment.

### Statistical Methods

Annual trend in the rate of emergency reoperations for MBS was analyzed by calculating the percentage of patients who underwent such reoperations for each year from 2017 to 2022. Preoperative demographics and medical comorbidities associated with a higher risk of emergency reoperations were identified using multivariable logistic regression. The covariates included in the regression model included age, sex, race, ASA class, highest body mass index (BMI) preoperatively, history of myocardial ischemia (MI), gastroesophageal reflux disease (GERD), hypertension (HTN), hyperlipidemia, diabetes, history of deep venous thrombosis (DVT), renal insufficiency, previous bariatric or foregut surgery, smoking, chronic obstructive pulmonary disease (COPD), history of PE, therapeutic anticoagulation, inferior vena cava filter, sleep apnea, surgical approach (laparoscopic, robotic, open), venous stasis, dialysis, type of MBS (SG, RYGB, BPD, and gastric band) and operative length. Subanalysis of these covariates was then performed for SG and RYGB to identify high-risk preoperative factors specific to these surgeries.

The nearest neighbor matching was then used to match 1:1 those patients who required emergency reoperations versus those who did not require emergency reoperations. Logistic regression distance using caliper 0.01 was utilized. The variables that were significantly different between the 2 cohorts were matched, which included age, race, sex, operative year, history of MI, highest BMI preoperatively, GERD, HTN, hyperlipidemia, surgical approach, renal insufficiency, dialysis, diabetes, COPD, type of bariatric surgery, venous stasis, sleep apnea, ASA class, history of DVT, and history of smoking within past year. Univariate analysis of postoperative outcomes within 30 days was then performed. All reported *P*-values were corrected for multiple hypothesis testing using the Benjamini-Hochberg correction. The analyses were performed using R: A Language and Environment for Statistical Computing, version 4.0.3.^13^

## RESULTS

During the study period, 1,223,256 patients underwent bariatric surgery, and 19,571 patients required reoperation, of which 9255 (<1%) were emergency reoperation. Overall, the rates of emergency reoperations have reduced significantly over the past 6 years from 0.84% to 0.60% (*P* < 0.001) (Table 1). BPD (2.0%) is associated with the highest rate of emergency reoperations, followed by RYGB (1.3%) (Table 2). About half the emergency reoperations (47%) occurred within the first 3 days after the surgery and two-thirds (67.8%) of the emergency reoperations occurred within the first week after surgery (Table 3). Some of the most common reasons for emergency reoperation included anastomotic or staple line leaks, GI tract bleeding and GI tract obstruction or stricture.

**TABLE 1.** Annual Trends in Emergency Reoperations From 2017 to 2022

Year	2017, N = 200,555	2018, N = 205,052	2019, N = 206,778	2020, N = 168,682	2021, N = 211,363	2022, N = 230,826	*P*-Value
Emergency reoperation	1680 (0.84%)	1833 (0.89%)	1838 (0.89%)	1139 (0.68%)	1378 (0.65%)	1387 (0.60%)	<0.001

**TABLE 2.** Trends in Emergency Reoperations by Type of Surgery

Characteristic	BPD*, N = 16,258	Gastric Band, N = 8154	RYGB**, N = 317,549	Sleeve, N = 770,100	*P*-Value
Emergency reoperation	332 (2.0%)	39 (0.5%)	4117 (1.3%)	3107 (0.4%)	<0.001

BPD indicates biliopancreatic diversion; RYGB, Roux-en-Y gastric bypass.

**TABLE 3.** Trends in Days Passed from Index Operation to Emergency Reoperation

Days to Reoperation	Number of Emergency Reoperations (%)
POD 0–3	4346 (47.0%)
POD 4–7	1928 (20.8%)
POD >7	2981 (32.2%)

POD indicates postoperative day.

The patients who underwent emergency reoperation were older and had a lower BMI compared with those who did not require emergency reoperation (Table 4). Greater proportion of males required emergency reoperation as compared with females among those who underwent MBS. Patients with comorbidities including history of DVT, therapeutic anticoagulation, renal insufficiency, and history of MI were at higher risk of requiring emergency reoperation (Table 4). Among the unmatched cohorts, death within 30 days was significantly higher in patients who underwent emergency reoperation as compared with those who did not require emergency reoperation (4.3% vs 0.07%, *P* < 0.001).

**TABLE 4.** Preoperative Characteristics of Patients Undergoing Emergency Reoperations

Characteristics	No Reoperation (N = 1,213,312)	Emergency Reoperation (N = 9249)	*P*-Value
Age	44.0 (36.3, 53.2)	48.3 (40.2, 57.1)	<0.001
Highest BMI pre-op	45.0 (40.8, 50.9)	44.3 (39.6, 50.7)	<0.001
Race			
Asian	6871 (0.6%)	44 (0.5%)	0.001
Black or African American	233,919 (22%)	1678 (20%)	
White	827,499 (77%)	6522 (79%)	
Surgical approach			
Laparoscopic	1,134,421 (94%)	8431 (91%)	<0.001
Open	5212 (0.4%)	217 (2.4%)	
Robot-assisted	60,886 (5.1%)	573 (6.2%)	
Dialysis	3592 (0.3%)	59 (0.6%)	<0.001
Venous stasis	9248 (0.8%)	124 (1.3%)	<0.001
History of DVT	21,232 (1.8%)	379 (4.1%)	<0.001
Sleep apnea	430,539 (36%)	3673 (40%)	<0.001
ASA class			
ASA I	3484 (0.3%)	30 (0.3%)	<0.001
ASA II	246,867 (21%)	1641 (18%)	
ASA III	904,769 (76%)	6918 (75%)	
ASA IV	42,131 (3.5%)	576 (6.3%)	
Renal insufficiency	6625 (0.6%)	120 (1.3%)	<0.001
Hyperlipidemia	268,344 (22%)	2543 (28%)	<0.001
Hypertension	537,599 (45%)	4699 (51%)	<0.001
Previous cardiac surgery	10,935 (0.9%)	185 (2.0%)	<0.001
History of myocardial infarction	13,526 (1.1%)	214 (2.3%)	<0.001
Gastroesophageal reflux	390,747 (33%)	4134 (45%)	<0.001
Smoker within 1 year	87,463 (7.3%)	857 (9.3%)	<0.001
Chronic obstructive pulmonary disease	15,874 (1.3%)	297 (3.2%)	<0.001
Sex			
Female	980,671 (99.30%)	7252 (0.73%)	<0.001
Male	219,492 (99.11%)	1965 (0.89%)	
IVC filter	3075 (0.3%)	951 (0.6%)	<0.001
Diabetes			
No	921,368 (77%)	6884 (75%)	<0.001
Yes	197,456 (16%)	1513 (16%)	
Yes, insulin-requiring	81,695 (6.8%)	824 (8.9%)	
History of pulmonary embolism	16,489 (1.4%)	302 (3.3%)	<0.001
Previous obesity/foregut surgery	156,688 (13%)	2667 (29%)	<0.001
Therapeutic anticoagulation	35,840 (3.0%)	583 (6.3%)	<0.001
Death within 30 days	859 (<0.1%)	402 (4.3%)	<0.001

IVC indicates inferior vena cava.

Multiple regression analysis of preoperative demographics associated with higher risk of emergency reoperation included older age and male sex. Patients with history of MI had 35% higher risk, sleep apnea had 59% higher risk, and previous surgery and a history of smoking both had 36% higher risk of emergency reoperations. History of DVT was associated with a 46% higher risk and therapeutic anticoagulation was associated with 34% higher risk of reoperation. The odds of requiring emergency reoperation were 3 times higher for BPD and 2 times higher for RYGB (Table 5).

**TABLE 5.** Multiple Regression Analysis of Preoperative Factors Associated With Emergency Reoperation

Characteristic	OR	95% CI	*P*-Value
Age			
20–40	Ref		
41–60	1.30	1.22–1.39	<0.001
61–75	1.49	1.36–1.63	<0.001
>75	1.31	0.69–2.21	0.4
Sex			
Female	Ref		
Male	1.19	1.11–1.26	<0.001
Race			
White	Ref		
American Indian or Alaska Native	0.90	0.62–1.24	0.5
Asian	1.09	0.78–1.48	0.6
Black or African American	0.98	0.92–1.04	0.5
Native Hawaiian or Other Pacific Islander	0.93	0.56–1.43	0.8
ASA class			
ASA I—Normal/healthy	Ref		
ASA II—Mild systemic disease	0.76	0.46–1.39	0.3
ASA III—Severe systemic disease	0.80	0.49–1.47	0.4
ASA IV—Severe systemic disease threat to life	1.00	0.60–1.85	>0.9
History of myocardial infarction	1.35	1.14–1.59	<0.001
Highest BMI pre-op			
35.0–39.9	Ref		
40.0–44.9	0.89	0.83–0.96	0.003
45.0–49.9	0.87	0.80–0.94	<0.001
50.0–59.9	0.82	0.76–0.89	<0.001
60.0 and above	0.91	0.81–1.01	0.078
Gastroesophageal reflux	1.17	1.11–1.23	<0.001
Hypertension	1.07	1.01–1.14	0.017
Hyperlipidemia	1.00	0.94–1.07	>0.9
History of DVT	1.46	1.26–1.69	<0.001
Renal insufficiency	1.45	1.12–1.83	0.003
Previous surgery	1.36	1.27–1.46	<0.001
Smoker	1.36	1.24–1.48	<0.001
Chronic obstructive pulmonary disease	1.59	1.38–1.83	<0.001
Sleep apnea	1.03	0.98–1.09	0.3
Surgical approach			
Laparoscopic	Ref		
Open	1.28	0.82–1.90	0.3
Robot-assisted	0.99	0.89–1.10	0.8
Diabetes	0.93	0.87–0.99	0.022
History of pulmonary embolism	1.31	1.11–1.54	0.001
Venous stasis	1.19	0.96–1.46	0.093
Dialysis	1.34	0.92–1.88	0.11
Therapeutic anticoagulation	1.34	1.19–1.51	<0.001
IVC filter	0.86	0.61–1.18	0.4
Surgery			
Sleeve	Ref		
BPD	3.30	2.88–3.76	<0.001
Gastric band	1.13	0.77–1.60	0.5
RYGB	2.25	2.11–2.39	<0.001
Operative length (min)			
<90	Ref		
90–179	1.41	1.32–1.50	<0.001
180–269	1.96	1.79–2.15	<0.001
270–359	3.21	2.76–3.71	<0.001
360–479	3.96	3.03–5.09	<0.001
480 and above	3.22	1.83–5.22	<0.001

CI indicates confidence interval; IVC, inferior vena cava; OR, odds ratio.

Analysis of preoperative comorbidities and demographics for patients undergoing SG showed similarly increased risk for older age and male sex. However, history of MI, HTN, and hyperlipidemia were not significant factors for requiring emergency reoperations in patients undergoing SG. Previous surgery and COPD were associated with a greater than 70% risk of emergency reoperations. History of PE and therapeutic anticoagulation were also associated with greater than 30% risk of emergency reoperations (Supplemental Table 1A, see https://links.lww.com/AOSO/A535). In the RYGB cohort, sex was not associated with a higher risk of emergency reoperations. Diabetes was protective against requiring emergency reoperations (odds ratio: 0.85, *P* < 0.001) (Supplemental Table 1B, see https://links.lww.com/AOSO/A535).

Given that the patients requiring emergency reoperations were generally older, with more comorbidities compared with those who did not require emergency reoperations (Table 4), patients in the 2 cohorts were matched to analyze the effect of emergency reoperations on postoperative outcomes within 30 days. Matching resulted in well-balanced cohorts of 7889 patients in both categories. Patients who underwent emergency reoperations were at greater than 50 times risk of cardiac arrest requiring cardiopulmonary resuscitation, unplanned ICU admission, prolonged ventilation postop, and organ space infection. Acute renal failure was significantly higher in those who required emergency reoperations (3.4% vs 0.1%, *P* < 0.001). Mortality within 30 days was 19 times higher in patients who required emergency reoperations compared with those who did not (3.48% vs 0.19%, *P* < 0.001). Myocardial infarction (0.44% vs 0.05%, *P* < 0.001), PE (1.75% vs 0.18%, *P* < 0.001), and pneumonia (0.15% vs 0.01%, *P* < 0.001) were also significantly higher in patients who required emergency reoperations (Table 6).

**TABLE 6.** Postoperative Outcomes Associated With Patients Requiring Emergency Reoperation

Characteristic	No, N = 7859	Yes, N = 7106	Difference	95% CI	*P*-Value
Death within 30 days	15 (0.19%)	247 (3.48%)	18.80	11.2–34.2	<0.01
Acute renal failure	9 (0.10%)	240 (3.4%)	30.50	15.8–67.5	<0.01
Cardiac arrest requiring CPR	3 (0.04%)	150 (2.11%)	56.40	18.9–275	<0.01
Myocardial infarction	4 (0.05%)	31 (0.44%)	8.60	3.04–33.6	<0.01
CVA	0 (0.00%)	27 (0.38%)	Inf	7.58–Inf	<0.01
PNA	1 (0.01%)	11 (0.15%)	12.20	1.77–523	0.01
PE	14 (0.18%)	124 (1.75%)	9.95	5.71–18.7	<0.01
Unplanned ICU admission	65 (0.83%)	2191 (30.83%)	53.40	41.6–69.5	<0.01
Prolonged ventilation post-op	8 (0.10%)	534 (7.51%)	79.70	40.1–186.0	<0.01
Wound disruption	13 (0.17%)	96 (1.35%)	8.26	4.61–16.1	<0.01
Deep incisional SSI	13 (0.17%)	149 (2.10%)	12.90	7.32–24.9	<0.01
Organ space infection	41 (0.52%)	1,515 (21.32%)	51.70	37.8–72.4	<0.01
Sepsis	3 (0.04%)	30 (0.42%)	11.10	3.45–56.9	<0.01
*Clostridium difficile*	11 (0.14%)	86 (1.21%)	8.74	4.64–18.2	<0.01
UTI	5 (0.06%)	6 (0.08%)	1.33	0.34–5.50	0.80
Dehydration requiring treatment	302 (3.84%)	483 (6.80%)	1.82	1.57–2.12	<0.01

CPR indicates cardiopulmonary resuscitation; CVA, cerebrovascular accident; PNA, pneumonia; SSI, surgical site infection; UTI, urinary tract infection.

## DISCUSSION

To our best knowledge, this is the first ever large database analysis of emergency reoperations after MBS in North America from 2017 to 2022. Overall, the rate of emergency reoperations has declined significantly (from 0.84% to 0.60%). Up to half the emergency reoperations occurred within postoperative days 0–3 and two-thirds occurred within a week from initial MBS. Some of the preoperative demographics and comorbidities of patients requiring emergency reoperations were older age, males, history of DVT, history of MI, GERD, therapeutic anticoagulation, and COPD, to name a few. Specifically for SG, the preoperative factors associated with higher risk of reoperations included older age, open surgery, GERD, smoking, COPD, history of DVT, and PE. The high-risk preoperative factors for gastric bypass were similar to factors for all bariatric procedures. Analysis of postoperative outcomes after matching patients who underwent emergency reoperation with those who did not undergo emergency reoperation showed higher rates of death, acute renal failure, unplanned ICU admissions, and organ space infections. Together, these findings provide insight into preoperative risk factors associated with emergent reoperations and the significantly worse perioperative outcomes, thus raising the need to follow the high-risk patients closely postoperatively.

The overall safety of bariatric surgery has improved over the years with 10-fold decrease in mortality since 1990s.^4,7,14^ This may explain the decreasing rates of emergency reoperations over the past 6 years in our study. Some of the perioperative factors associated with reoperation in prior studies include dialysis, history of bleeding disorders, and longer operative periods, which is similar to our findings.^11^ Recent analysis of emergent reoperations after MBS utilizing the Michigan Bariatric Surgery Collaborative showed that older age, HTN, and longer operative times were associated with reoperation <24 hours after SG, which is consistent with our findings.^12^ Analysis of NSQIP database from 2007 to 2009 also showed that gastric bypass, dialysis, bleeding disorder, and longer length of operation were associated with increased risk of return to the operating room.^11^ Another NSQIP database study of patients undergoing SG or RYGB from 2011 to 2015 showed that smoking, COPD, HTN, male sex, and old age were patient-specific independent factors predictive of reoperations.^9^

Our study showed that the risk of mortality within 30 days was almost 19 times higher in patients requiring emergent reoperations. This is slightly higher than the mortality reported in prior studies evaluating the outcomes for reoperation after bariatric surgery, ranging from 1.4% to 2.2%.^9,11^ Prior studies evaluating the outcomes of emergency versus elective surgeries have shown more than 2 to 5 times higher mortality rates for emergency surgeries compared with elective surgeries.^15–17^ This is likely due to acute systemic dysfunction contributing to the emergency nature of the surgery and the poor nutritional status, which is often magnified postoperatively in patients who have undergone MBS due to malabsorption. The morbidity is also significantly higher in emergent surgeries compared with elective surgeries for similar reasons.^18^ This is reflected in the postoperative outcomes for emergency reoperations after MBS in our study. The emergent nature of the reoperations limits the ability to optimize the patients preoperatively.

A major limitation of our study is its retrospective nature, which limits the evaluation of the factors uncaptured in the database. Intraoperative technical, physiologic, and anatomical challenges are not captured in the MBSAQIP database, which may contribute significantly to the need for emergent reoperations. We have tried to mimic the randomized control trial by balancing the confounding factors through propensity score matching in those who required emergency reoperation versus those who did not. Additionally, the study is limited to outcomes within 30 days. It is unknown how the emergent reoperations affect the remission of metabolic comorbidities and weight loss. Further studies evaluating the effect of these emergent reoperations on long-term effects of MBS are warranted.

## CONCLUSION

Emergent reoperations after MBS have significantly worse morbidity and mortality. Recognition of high-risk preoperative factors can help optimize the modifiable factors preoperatively and facilitate closer monitoring of vulnerable patients.

## Supplementary Material


