# Supplementary material for: National Analysis of Emergent Reoperations After Metabolic Bariatric Surgery Over 6 Years
Source: Ann Surg Open. 2025 Sep 23;6(4):e611. doi: 10.1097/AS9.0000000000000611 (PMC12727272; doi:10.1097/AS9.0000000000000611)
Supplement: Supplementary file 1 [file as9-6-e611-s001.pdf]

Supplementary Table 1: Multiple Regression of Pre-operative Factors Associated with Emergency Reoperation by Type of Metabolic-Bariatric Surgery

Table 1A: Multiple Regression of Pre-operative Factors Associated with Emergency Reoperation for Sleeve Gastrectomy

**Characteristic**	**OR**	**95% CI**	**p-value**
Age			
20-40	—	—	
41-60	1.26	1.14, 1.39	<0.01
61-75	1.49	1.29, 1.71	<0.01
>75	0.56	0.14, 1.47	0.30
Sex			
Female	Ref		
Male	1.37	1.25, 1.50	<0.01
Race			
White	—	—	
Asian	1.37	0.87, 2.04	0.14
Black or African American	0.92	0.84, 1.02	0.11
ASA Class			
ASA I - Normal/Healthy	—	—	
ASA II - Mild systemic disease	0.54	0.30, 1.14	0.07
ASA III - Severe systemic disease	0.59	0.32, 1.23	0.11
ASA IV - Severe systemic disease threat to life	0.83	0.44, 1.75	0.60
History of myocardial infarction	1.20	0.91, 1.56	0.2
Highest BMI pre-operatively			
35.0-39.9	—		
40.0-44.9	0.86	0.77, 0.97	0.01
45.0-49.9	0.87	0.77, 0.98	0.03
50.0-59.9	0.82	0.72, 0.93	0.01
60.0 and above	0.89	0.75, 1.05	0.20
Gastroesophageal reflux	1.20	1.11, 1.31	<0.01
Hypertension	1.09	1.00, 1.19	0.06
Hyperlipidemia	1.00	0.90, 1.11	>0.90
History of DVT	1.56	1.24, 1.95	<0.01
Renal insufficiency	1.64	1.17, 2.25	0.01
Previous Surgery	1.70	1.48, 1.94	<0.01

Smoker	1.26	1.10, 1.44	<0.001
Chronic obstructive pulmonary disease	1.71	1.37, 2.12	<0.001
Sleep apnea	1.01	0.93, 1.10	0.8
Surgical approach			
Laparoscopic	—	—	
Open	4.35	1.00, 12.8	0.02
Robot-assisted	0.91	0.77, 1.07	0.30
Diabetes	1.02	0.93, 1.12	0.70
History of Pulmonary Embolism	1.33	1.02, 1.71	0.03
Venous stasis	1.26	0.91, 1.70	0.14
Dialysis	1.51	0.99, 2.24	0.05
Therapeutic Anticoagulation	1.38	1.15, 1.64	<0.01
IVC filter	0.70	0.39, 1.17	0.20
Operative length (in mins)			
<90	—	—	
90-179	1.44	1.32, 1.57	<0.01
180-269	2.58	2.06, 3.20	<0.01
270-359	5.36	3.26, 8.29	<0.01
360-479	4.85	1.66, 11.0	<0.01
480 and above	2.64	0.65, 6.94	0.10

Table 1B: Multiple Regression of Pre-operative Factors Associated with Emergency Reoperation for RYGB

**Characteristic**	**OR**	**95% CI**	**p-value**
Age			
20-40	—	—	
41-60	1.27	1.17, 1.39	<0.01
61-75	1.42	1.25, 1.61	<0.01
>75	2.20	1.04, 4.07	0.02
Sex			
Female	—	—	
Male	1.05	0.96, 1.15	0.30
Race			
White	—	—	
Asian	0.80	0.45, 1.30	0.40
Black or African American	1.05	0.96, 1.15	0.30
ASA Class			
ASA I - Normal/Healthy	—	—	
ASA II - Mild systemic disease	1.05	0.44, 3.40	>0.90
ASA III - Severe systemic disease	1.07	0.45, 3.46	0.90
ASA IV - Severe systemic disease threat to life	1.11	0.46, 3.62	0.80
History of Myocardial infarction	1.35	1.06, 1.69	0.01
Highest BMI pre-op			
35.0-39.9	—	—	
40.0-44.9	0.91	0.82, 1.01	0.08
45.0-49.9	0.83	0.75, 0.93	0.01
50.0-59.9	0.79	0.71, 0.89	<0.01
60.0 and above	0.90	0.77, 1.05	0.20
Gastroesophageal reflux	1.14	1.07, 1.23	<0.01
Hypertension	1.07	0.99, 1.16	0.10
Hyperlipidemia	1.01	0.93, 1.10	0.80
History of DVT	1.38	1.13, 1.68	0.01
Renal insufficiency	1.31	0.88, 1.88	0.20
Previous Surgery	1.17	1.07, 1.29	<0.01
Smoker	1.45	1.28, 1.64	<0.01
Chronic obstructive pulmonary disease	1.52	1.24, 1.85	<0.01
Sleep apnea	1.02	0.95, 1.10	0.60
Surgical approach			
Laparoscopic	—	—	
Open	0.96	0.48, 1.71	0.90
Robot-assisted	1.07	0.92, 1.23	0.40
Diabetes	0.85	0.78, 0.92	<0.01

History of PE	1.26	1.01, 1.57	0.04
Venous stasis	1.18	0.87, 1.56	0.30
Dialysis	0.62	0.22, 1.37	0.30
Therapeutic Anticoagulation	1.40	1.18, 1.65	<0.01
IVC filter	1.04	0.65, 1.57	0.90
Operative length (in minutes)			
<90	—	—	
90-179	1.28	1.17, 1.40	<0.01
180-269	1.71	1.53, 1.92	<0.01
270-359	3.17	2.68, 3.74	<0.01
360-479	3.70	2.72, 4.94	<0.01
480 and above	2.99	1.41, 5.53	0.01
